# The efficacy of ilioinguinal and iliohypogastric nerve block for postoperative pain after caesarean section

**Published:** 2010

**Authors:** Melike Sakalli, Ayşegül Ceyhan, Hale Yarkan Uysal, Işin Yazici, Hülya Başar

**Affiliations:** a Anaesthesiologist, Ankara Training and Research Hospital, Anaesthesiology and Reanimation Clinic, Ankara, Turkey; b Associate Professor of Anaesthesiology, Ankara Training and Research Hospital, Anaesthesiology and Reanimation Clinic, Ankara, Turkey; c Resident in Anaesthesiology, Ankara Training and Research Hospital, Anaesthesiology and Reanimation Clinic, Ankara, Turkey; d Associate Professor of Anaesthesiology, Head of Department, Ankara Training and Research Hospital, Anaesthesiology and Reanimation Clinic, Ankara, Turkey

**Keywords:** Caesarean Section, Postoperative Analgesia, Ilioinguinal and Iliohypogastric (II-IH) Nerve Block

## Abstract

**BACKGROUND:**

The effect of ilioinguinal and iliohypogastric (II-IH) nerve block on postoperative pain is well documented when performed before Caesarean section (CS) but the efficacy remains unclear when performed after the surgical procedure. The aim of this study is to investigate the effect of II-IH nerve block on postoperative pain and analgesic consumption in patients when performed after CS.

**METHODS:**

Sixty ASA I- II patients, scheduled for elective CS were included in the study. After general anaesthesia, patients were allocated into 2 groups randomly. In group I bilateral II-IH block has been performed after the skin closure, with 10 ml of 0.5% ropivacaine on each side. In group II sham block had been performed. For postoperative analgesia all patients received tramadol via i.v patient controlled analgesia. Visual analogue scale (VAS) scored tramadol consumption and side effects.

**RESULTS:**

The mean VAS scores in II-IH block group were significantly lower than in sham block group at 6^th^, 8^th^, 12^th^, 24^th^ hours at rest (p < 0.05) and at 6^th^, 8^th^ hours with movement (p < 0.05). Tramadol usage in II-IH block group was significantly less than in sham block group at all estimated time intervals (p < 0.05). Total tramadol consumption was 331 ± 82 mg in II-IH block group and 622 ± 107 mg in sham block group (p < 0.05).

**CONCLUSIONS:**

It was observed that II-IH nerve block when performed after the surgery may reduce analgesic consumption after CS.

Caesarean section (CS) has been one of the most frequently performed major surgical interventions, and causes severe postoperative pain. Regional anaesthesia is mostly preferred for CS and it is advantageous in the early postoperative period due to residual analgesia. On the other hand, general anaesthesia is also used, which necessitates intramuscular or intravenous analgesics, including opioid drugs. Opioid drugs are effective in reducing postcaesarean pain but side effects such as nausea, vomiting and sedation limit their use.^1^

Childbirth is an emotion-filled event and the mother needs to bond with her newborn as early as possible. Any intervention that leads to improvement in pain relief is worthy of investigation. Preoperative ilioinguinal and iliohypogastric (II-IH) nerve blocks has been widely used to provide analgesia for children and adults undergoing surgery for inguinal hernia repair,^2^–^5^ and also for postoperative analgesia after CS in parturients.^6^–^8^ The beneficial effects of II-IH nerve block on postoperative pain after CS is well documented as a preemptive technique^6^,^7^ but the efficacy remains unclear when performed after the surgical procedure.^8^,^9^ Therefore, this double blind, prospective and randomized study was undertaken to investigate the efficacy of II-IH nerve block on postoperative pain and analgesic consumption in patients when performed after CS.

## Methods

After obtaining approval from Ministry of Health, Ankara Training and Research Hospital Ethics Committee, and written informed consent; ASA I-II, 64 term parturients, scheduled for elective caesarean operation requiring general anaesthesia were included in a double-blind, randomized clinical trial in 4 months period. Exclusion criteria were as follow: preeclampsia, eclampsia, renal, hepatic or cardiac disorders, a history of allergy to the study drugs, a history of chronic pain and chronic analgesic usage or any infection on the nerve block area.

Patients were premedicated with 150 mg ranitidine or 20 mg famotidine orally the night before and thirty minutes prior to induction of anaesthesia, the patient received 30 ml of sodium citrate 0.3 mol.L ^−1^orally. Standard monitoring including ECG, pulse- oximetry and non- invasive blood pressure were applied to each patient in the operating room. Uterine displacement was achieved by tilting the operating table to the left. After insertion of a wide-bore intravenous (iv) canule, the patient’s fluid defisit was calculated to be replaced over 3 hours with lactated Ringer’s solution and maintenance fluid was calculated according to patients’ weight. Following 5 minute of pre-oxygenation, rapid sequence of induction was performed with thiopental 4 mg kg^−1^ and 1 mg kg^−1^ rocuronium intravenously. Cricoid pressure was performed from loss of consciousness to confirmation of tracheal cuffed tube position. After tracheal intubation, the patients’ lungs were ventilated with desflurane 3% and nitrous oxide 50% in oxygen, maintaining endtidal carbon dioxide (ETCO_2_) between 30 and 35 mmHg. Fentanyl iv 1 μg kg^−1^ was administered for intraoperative analgesia after the clamping of the umblical cord.

Patients were randomly allocated into two equal groups after induction of general anaesthesia with a closed envelope technique. After antagonization of the neuromuscular block with iv neostigmine 0.04 mg kg^−1^ and 0.02 mg kg^−1^ atropine, before extubation, II-IH nerve block was performed in group I (II-IH block) and patients received a sham block with saline at the corresponding puncture site in group II (sham block). The study solutions were prepared by one of the researchers who was not involved in the study. All blocks were performed by the same author in the study who was blinded to the used drugs, using the technique described by Bell et al.^8^ Approximately 2 cm medial and superior point of the anterior superior iliac spine was marked. A 22G needle was advanced to the skin and after piercing the external oblique fascia, one sixth of 10 ml of 5% ropivacaine (Naropin^®^, AstraZeneca AB, Södertalje, Sweden) was injected after a negative aspiration test. Then the needle was advanced until the loss of resistance was felt between internal oblique muscle and transverse muscle. Another one sixth of the local anaesthetic solution was injected. The needle was withdrawn and this procedure was repeated by changing the needle to medially and then laterally at angles of 15° at the same horizontal plane in a fanlike pattern. The same procedure was performed on the contralateral side and a total volume of 20 ml of local anaesthetic was administered for both sites of injection. Sham block with same amount of saline was performed using the technique described by Bell et al^8^ in the sham block group.

At the end of the operation the trachea was extubated and all patients were transferred to the post- anaesthesia care unit (PACU). At the PACU, pain and its intensity was assessed by an anaesthesiologist unaware of the group assignment when patients have been conscious and have communicated verbally. All patients received intravenous tramadol via patient controlled analgesia (PCA) pump for postoperative analgesia at the PACU. PCA device was set to deliver a 50 mg loading dose of tramadol with a bolus dose of 25 mg and a lock out time of 15 minutes and 4 hour limit of 300 mg. Pain was assessed at rest and with movement (turning from side to side) by using visual analogue scale (VAS) ranging from 0 (no pain) to 10 cm (intolerable pain) at the PACU in 0, 2, 4, 6, 8, 16, 20, 24 hours postoperatively. Patients who had a VAS of 3 or more in spite of iv- tramadol PCA (throughout the study period) received 0.5 mg kg^−1^ iv meperidin as a rescue analgesia. At the same time intervals; heart rate, blood pressure and respiratory rate were assesed. Adverse events such as nausea and vomiting and sedation were also recorded at the same time intervals. Nausea was defined as an unpleasant feeling associated with inclination to vomit, and vomiting was defined as the forceful ejection of gastric contents through the mouth. Retching was also recorded as vomiting. Patients who had nausea and vomiting received iv 10 mg metoclopramide. Sedation was assessed using a 4- point scale (0 = fully awake, 1 = awake but drowsy, 2 = sleeping, but arousable by light thouch or speech, 3 = sleeping, not arousable).

At the end of the study, total tramadol consumption and rescue analgesia requirements were also recorded.

### 

#### Statistical Analysis

Primary end point of the study was to evaluate 30% decrease in analgesic consumption between the two groups at estimated time intervals postoperatively. A sample size of at least 25 patients per group was required to detect this difference with a power of 90% at the 5% significance level. All statistical analyses were performed using SPSS software (Statistical Package for Social Sciences) for Windows, version 11.5. Data are presented as mean and standard deviation. Means were compared by using Student’s t or Mann Whitney U tests, where appropriate. Chi- square or Fisher’s Exact tests were used for categorical comparisons. Bonferroni Correction was applied for between group comparisons of repeated variables. A p value less than 0.05 was considered statistically significant.

## Results

Sixty four patients were included in the study. Four patients were excluded from the study because of blood aspiration with a negative aspiration test at the nerve block area while performing the II-IH block. No significant differences with regard to demoghraphic data, duration of surgery or gestational week could be observed among the study groups (Table 1).

**Table 1:** Patients characteristics, gestational week and surgical times. Data are expressed as mean ± SD.

Variable	II-IH block (n = 30)	Sham block (n = 30)
Age (year)	26.27 ± 2.72	27.13 ± 4.20
Weight (kg)	72.93 ± 8.12	77.30 ± 10.20
Height (cm)	163.80 ± 4.96	164.03 ± 4.56
Duration of surgery (minute)	36.97 ± 6.48	40.83 ± 9.38
Gestational week (week)	39.05 ± 0.44	39.11 ± 0.56

There were no significant differences in systolic and diastolic blood pressure values and heart rate values between groups and within-group comparisons (p > 0.05).

**Figure 1:**
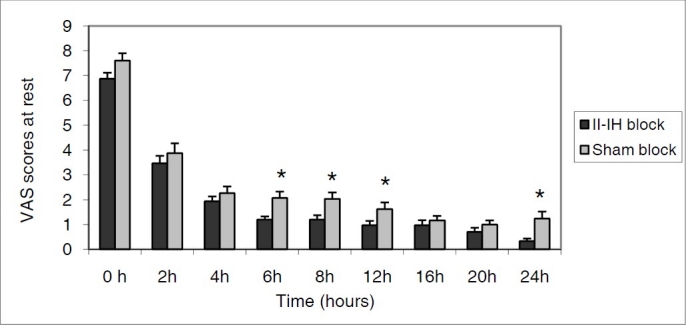
Postoperative VAS scores at rest. Data are expressed as mean ± SD. * Statistically significant difference between II-IH block and sham block group (p < 0.05).

**Figure 2:**
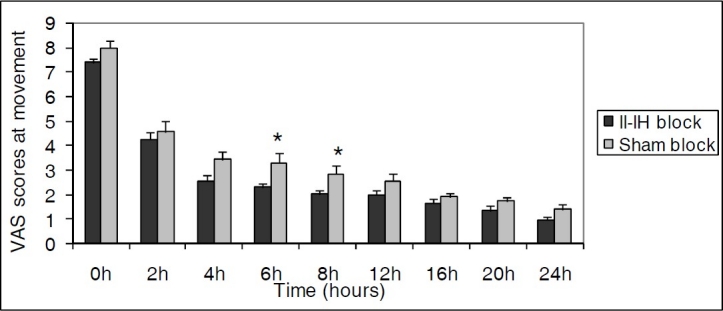
Postoperative VAS scores at movement. Data are expressed as mean ± SD. * Statistically significant difference between II-IH block and sham block group (p < 0.05).

The mean VAS scores in II-IH block group were significantly lower than in sham block group at 6^th^, 8^th^, 12^th^, 24^th^hours at rest (p < 0.05) (Figure 1) and at 6^th^, 8^th^ hours with movement (p < 0.05) (Figure 2).

**Figure 3:**
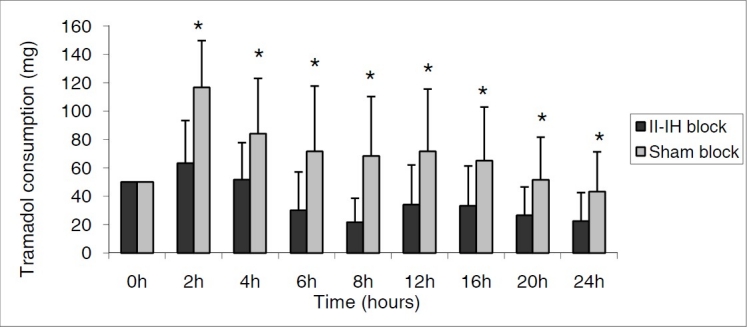
Tramadol consumption (mg) at estimated time intervals. Data are expressed as mean ± SD. * Statistically significant difference between II-IH block and sham block group (p < 0,006). (Applied Bonferroni Correction)

In II-IH block group, tramadol usage was apparently lower than in sham block group at all estimated time intervals throughout the study (p < 0.05) (Figure 3). At the end of the study period; the mean total PCA tramadol dose was nearly twice as high in sham block group (622 ± 107 mg) compared to II-IH block group (331 ± 82 mg) (p < 0.05).

Seven patients needed additional meperidine in the sham block group, but only 1 patient in the II-IH block group needed it (p > 0.05).

No statistically difference was found between the two groups with regard to side effects such as nausea, vomiting and sedation (p > 0.05) (Table 2).

**Table 2:** Percentage of patients who had side effects as nausea, vomiting and sedation at estimated time intervals. Data are presented as percent.

Nausea	At the PACU (0)	2^nd^ h	4^th^ h	6^th^ h	8^th^ h	12^th^ h	16^th^ h	20^th^ h	24^th^ h
II-IH block	6.7%	0%	0%	0%	13.3%	10.0%	6.7%	3.3%	0%
Sham block	23.3%	16.7%	16,7%	16,7%	13.3%	13.3%	3.3%	0%	0%
**Vomiting**									
II-IH block	0%	0%	0%	0%	0%	0%	0%	0%	0%
Sham block	0%	16,7%	10.0%	6.7%	6.7%	3.3%	3.3%	0%	0%
**Sedation**									
II-IH block	0%	3.3%	0%	3.3%	3.3%	0%	0%	0%	0%
Sham block	6.7%	10.0%	10.0%	6.7%	6.7%	6.7%	3.3%	0%	0%

## Discussion

The present data indicate that II-IH nerve block, when performed after surgery, is effective in reducing postoperative pain and analgesic consumption after CS.

Lower segmental CS is performed by Pfannenstiel incision which lies on L1-L2 dermatoms. Sensory innervation of L1-L2 dermatoms is accomplished by ilioinguinal and iliohypogastric nerves. Block of these nerves enables somatic pain relief in CS operations, but is ineffective for visceral pain, as viscerae are innervated by nerve roots from T10-L1 segments.^6^ II-IH block have been reported to decrease the need for postoperative analgesics at rates of 35% to 78%, depending on the operative procedure or anatomical variations.^10^ Bunting et al performed II-IH nerve block technique on CS patients with 0.5% bupivacaine and found out that II-IH block group had lower pain scores and lower analgesic requirements compared to no- block group.^6^ In another study, Ganta et al reported lower VAS scores and less need for additional analgesia with II-IH block in patients after CS under general anaesthesia.^7^

Huffnagle et al reported that pain control and consumption of opioid drugs during the first postoperative day, by bilateral II-IH block after the spinal anaesthesia but before the incision did not differ from the sham block group, for elective CS operations. In the same study, patients who received bilateral II-IH nerve block after caesarean delivery had more pain than those who had sham block.^9^ Some data suggest that postoperative nerve block or wound infiltration does not reduce postoperative pain, but may instead, increase pain. An explanation may be the inhibition of the body’s adaptive responses to that pain caused by central or peripheral nerve blocks placed after the onset of a painful stimulus. As a result, when the local anaesthetic effects wane, the returning, unmodulated pain, is more severe than when the patient had received no anaesthetic intervention.^11^ In contrast to hypothesis explained above, Bell et al^8^ reported significantly reduced amount of iv morphine use by the ilioinguinal-iliohypogastric nerve block (performed after wound closure) in patients during the 24 hour following caesarean delivery. In the present study concordant results with Bell et al^8^ were obtained; patients who received II-IH nerve block after surgical intervention revealed lower pain scores than control group. Also consumption of tramadol of the patients with the II-IH block were found to be apparently low for the first postoperative day.

Ilioinguinal and iliohypogastric nerve block had been performed with ropivacaine in numerous studies. Solutions of ropivacaine 5- 10 mg ml^−1^ (0.5%- 1%) are likely to have wide clinical application compared to bupivacaine^12^,^13^ due to their lower systemic potential for central nervous system and cardiovascular toxicity.^14^,^15^Wulf et al reported that 60- 70 ml of 0.5% ropivacaine had safe and satisfactory results for the postoperative analgesia after in-guinal hernia repair.^16^ Dalens et al evaluated the optimal doses of ropivacaine for II-IH nerve blockade in pediatric patients and concluded that 3 mg kg^−1^ was sufficient with concentrations of 0.5%.^17^ In this study, ropivacaine was preferred to other local anaesthetics for its lower central nervous system and cardiovascular toxicity.

Sajedi et al evaluated inguinal field block when performed pre and post incision in pediatric patients undergoing herniorrhapy and concluded that pre-surgical infiltration of local anaesthetic in the surgical field is a useful method in alleviation of postoperative pain and analgesic consumption.^18^ The lack of a preemptive II-IH block group may be considered as one of the limitations in this study. That’s because the present study focused only on the efficacy of II-IH block performed after surgery and it was not prefered to perform II-IH nerve block before caesarean delivery because of the difficult manipulation of the block in the area of the gravid uterus. Another reason was to avoid prolongation of time between anaesthesia induction and uterus incision and effect on fetus. Further, obvious differences in the VAS scores at all estimated time intervals between the II-IH block group and sham block group could not be obtained. Especially at admission to PACU, it was expected that the VAS scores in II-IH block group to be lower than in sham block group but this was not observed. It is believed that this may be due to the early assessment of pain and the block needed much time to work. Also, lack of assesment of pain in shorter duration of time intervals at PACU may have contributed to the minimal differences between groups in VAS scores at the early period. For further time intervals, adequate analgesia, that is provided by the PCA pump and rescue analgesic medication in the sham block group, may explain the lack of significant differences in VAS scores throughout the study period.

The analgesic effects of weak opioids are reported to be equivalent to potent opioids when used intravenously for PCA with less side effects.^19^ Tramadol has been used extensively in the adult surgical population and is known to be safer regarding respiratory depression.^20^ But side effects such as nausea and vomiting are common in clinical application due to the opioid agonist effect of tramadol.^21^ Although tramadol use was found to be higher in sham block group, no statistically difference was detected in postoperative nausea and vomiting in this study. This may be because of the small sample size but it is considered that this might be because of the application of intravenous tramadol via PCA device instead of intermittent high bolus doses for postoperative analgesia.

Pharmacokinetically, breast milk is supposed to be a separate compartment into which the drug is excreted- mainly by passive diffusion. It is estimated that 0.1% of the administered dose of tramadol passes into breast milk.^22^ At the end of the present study total tramadol consumption was found to be 331 ± 82 mg in the II-IH block group, and 622 ± 107 mg kg^−1^ in the sham block group. It was examined that in the early postoperative hours the tramadol usage in II-IH group was very lower than the sham group. This significantly reflects the efficacy of II-IH nerve block especially on early postoperative pain.

## Conclusions

The application of II-IH nerve block is considered to be advantageous after CS operations regarding the unwanted effects of analgesics that passes into breast milk in newborns. By this technique, the dose of tramadol passing into breast milk is lessened as total systemic analgesic dose is reduced.

In the present study it was observed that II-IH nerve block, performed after CS operations under general anaesthesia, increased the quality of pain control in the postoperative period and apparently decreased the consumption of tramadol. Therefore it is advocated that II-IH nerve block, performed after the operation, is a preferrable technique for the pain control after CS.
